# Draft Genome Sequence of *Klebsiella michiganensis* 3T412C, Harboring an Arsenic Resistance Genomic Island, Isolated from Mine Tailings in Peru

**DOI:** 10.1128/genomeA.00611-17

**Published:** 2017-07-13

**Authors:** Robert Ccorahua-Santo, Miguel Cervantes, Yerson Duran, Mac Aguirre, Claudia Marin, Pablo Ramírez

**Affiliations:** Laboratory of Molecular Microbiology and Biotechnology, Faculty of Biological Science, Universidad Nacional Mayor de San Marcos, Lima, Peru

## Abstract

An arsenic resistance genomic island in the bacterium *Klebsiella michiganensis* 3T412C was isolated from mine tailings from Peru. This genomic island confers adaptation to extreme environments with high concentrations of arsenic. Isolate 3T412C contained a complete set of genes involved in resistance to arsenic. This operon is surrounded by putative genes for resistance to other heavy metals.

## GENOME ANNOUNCEMENT

Recent studies have revealed that both horizontal gene transfer (HT) and genomic islands (GIs) can confer selective advantages to bacteria (1). Multiple mechanisms have evolved for cellular defense against arsenic, and the genes involved are taxonomically widespread and subject to HT (2, 3). The general efflux detoxification pathway involves the reduction of arsenate to arsenite, and then subsequent expulsion of arsenic from the cell through arsenite-specific transporters (4, 5). The efflux system consists of an arsenate reductase (ArsC) (6), an arsenite-specific efflux pump (ArsB) (7), ATPase (ArsA) that couples with ArsB for the expulsion of arsenite from cells, the regulatory elements ArsR and ArsD, and a gene, *arsH*, of unknown function (2). The genes sufficient for a complete efflux pathway were previously identified in *Prochlorococcus* genomes (8). This efflux detoxification is believed to be the major arsenic detoxification strategy for *Prochlorococcus* and other species with the same operon configuration (3).

Here, we report the draft genome sequence of *Klebsiella michiganensis* strain 3T412C, which was isolated from surface water from a mine water treatment operation in Trujillo, Peru. The draft genome sequence was determined using Illumina sequencing, and assembly with SPAdes version 3.10 was carried out with *k*-mer values increasing from 51 to 71 (9). Additionally, reads were utilized for contig extension and gap repairing with ABACAS and IMAGE, respectively (10). The quality of the assemblies was verified with QUAST software (11). Default parameters were used. Finally, 187 contigs were submitted to GenBank. Preliminary gene prediction and annotation were performed with the Prokka tool (12). The existence of GIs was confirmed with IslandViewer software, a predictor of GIs that integrates three methods: IslandPick, IslandPath-DIMOB, and SIGI-HMM (13). The number of contigs was 120, and the *N*_50_ was 136,825 bp. The draft genome of 3T412C comprised 6,208,338 bp, with a G+C content of 55.71%.

We found that the genome contained a GI with an operon for arsenic resistance. Within the operon, the configuration of genes was the same as that for *Prochlorococcus* spp., in the following order: ArsA, ArsC, ArsB, ArsA, the operon repressor ArsD, and the regulatory element ArsR. This operon is surrounded by other putative resistance proteins for copper, cobalt, cadmium, mercury, lead, and zinc.

After evaluation of the persistent operon in the genome, we evaluated the bacterium’s tolerance of heavy metals. The growth of strain 3T412C was not affected by an arsenic concentration as high as 28 mM (arsenite, NaAsO_2_ from Merck) in the medium nonenriched LB at pH 7. In contrast with other reports on *Klebsiella* spp., this is the strain with the highest resistance potential (14).

### Accession number(s).

This whole-genome shotgun project for *K. michiganensis* strain 4T312C has been deposited at DDBJ/ENA/GenBank under the accession number MPJL00000000.
